# Computer tomographic analysis of anatomic characteristics of the ulna – essential parameters for preshaped implants

**DOI:** 10.1371/journal.pone.0232988

**Published:** 2020-05-21

**Authors:** Johannes Christof Hopf, Andreas Jähnig, Tobias Jorg, Ruben Sebastian Westphal, Daniel Wagner, Pol Maria Rommens

**Affiliations:** 1 Department of Orthopedics and Traumatology, University Medical Center, Mainz, Rhineland-Palatinate, Germany; 2 Department of Radiology, University Medical Center, Mainz, Rhineland-Palatinate, Germany; 3 Institute of Medical Biostatistics, Epidemiology and Informatics, University Medical Center, Mainz, Rhineland-Palatinate, Germany; Assiut University Faculty of Medicine, EGYPT

## Abstract

**Purpose:**

Anatomically preshaped implants are needed for exact restoration of the anatomy after fractures of the proximal ulna and ulnar shaft, which enables a good functional outcome. Aim of this computed tomographic analysis was to identify specific characteristics of the ulna. The data serve for the development of a new intramedullary implant for stabilisation of proximal and diaphyseal ulna fractures.

**Methods:**

With a standardized research method 100 CT scans of the ulna were evaluated regarding anatomic parameters like width of the medullary canal, proximal ulna dorsal angulation and varus angulation. Also, correlations of these parameters were analyzed statistically.

**Results:**

The mean proximal ulna dorsal angulation (PUDA) was 6.4° (SD 2.8°), while the mean varus angulation of the proximal ulna was 12.4° (SD 3.3°). The length of the ulna bone was 253.6 mm (SD 19.9 mm) on average. The average minimum diameter of the medullary canal was 4.2 mm (SD 1.1 mm) located at 141.3 mm (SD 19.7 mm) from the olecranon tip. There is a positive correlation between age and minimum diameter in our patient cohort (p< 0.001).

**Conclusion:**

Our study described the anatomy of the proximal ulna and the ulna shaft with a reproducible research method in a representative patient cohort. The knowledge of the evaluated anatomic parameters can lead to an improvement of any implant design for the fixation of proximal and diaphyseal ulna fractures.

## Introduction

Exact restoration of forearm anatomy in case of forearm fractures in adults is crucial for a good functional outcome [1,2]. Therefore, in-depth knowledge of the anatomy and its variabilities is mandatory. The anatomy of the ulna was described in several cadaveric studies as well as computed tomography morphometric analyses [3–5]. A dorsal and a varus angulation of the ulna is described in the proximal metaphysis. This leads to a complex anatomy with two joints for elbow and forearm motion and a wide individual variability [6–8].Especially the restoration of the proximal ulna dorsal angulation (PUDA) seems to have an impact on the functional outcome[9,10].

In case of displaced fractures of the ulna surgical treatment is actually done with open reduction and internal fixation [11,12]. Modern anatomical plates for ulna osteosynthesis are preshaped to match the specific anatomy and to allow precise reduction and stable fixation [13–15]. Intramedullary implants are not frequently used for these fractures, although the principle of intramedullary fixation showed good biomechanical and clinical outcomes on many other anatomic regions [16,17]. Anatomically preshaped nails with feasible instruments and suitable locking options are not available on the market, so theoretical advantages of intramedullary fixation as less periosteal stripping and preservation of the fracture hematoma are not used at the forearm [18,19]. Especially the diameter of the medullary canal is an important anatomical parameter, which must be considered prior to an intramedullary osteosynthesis. Most anatomic studies of the ulna do not analyse representative patient cohorts, which limits the significance of their results.

The aim of this study was to describe important anatomic characteristics of the ulna in a representative patient cohort and its subgroups. One hundred CT scans of the ulna were analysed with special interest on relevant anatomic landmarks. The data will be used for the development of a preshaped intramedullary implant for the proximal ulna and the ulnar shaft [20].

## Materials and methods

We analysed Computed-tomography (CT) scans of the forearm with intact ulna and radius bones, as well as intact elbow und wrist joints with 1mm slice thickness of one hundred individuals (60 males, 40 females, mean age 63.1 years (SD 19.8)). All CT-scans were angiographic CT scans of the whole arm in upright position in angiological patients. Scans with a prior ulna fracture or bony pathology other than osteopenia were excluded beforehand. Philips brilliance iCT (128 slices) and Philips brilliance CT (64 slices) scanners (Philips, Amsterdam, Netherlands) were used. The sample size was calculated for an estimated standard deviation of 0.192mm of the diameter of the medullary canal. A sample size of 89 CT scans was required for a 95% confidence interval half-width of 0.04mm. Gender and age distribution of the selected individuals were selected to match the epidemiological data of the Federal Statistical Office of Germany for ulna shaft fracture for the year 2017 [21]. The individuals in the study had similar age and gender distribution as the ulna fracture cohort according to the Federal Statistical Office (Table 1) [21]. No identifying information was used in this study. The CT data was pseudonymized before analysation. No ethics statement was required for this study.

**Table 1:** Gender and age distribution.

Parameters	Our study	Expected value
**Gender distribution [male/female]**	60/40	60/40
**Age distribution**		
18–44 years	21	30
45–64 years	28	26
> 64 years	51	44

Gender and age distribution of our patient collective in comparison to the expected values from the epidemiological data of the German Federal Statistical Office 2017 of forearm fractures and isolated ulna shaft fractures

### Measurements

**Fig 1:**
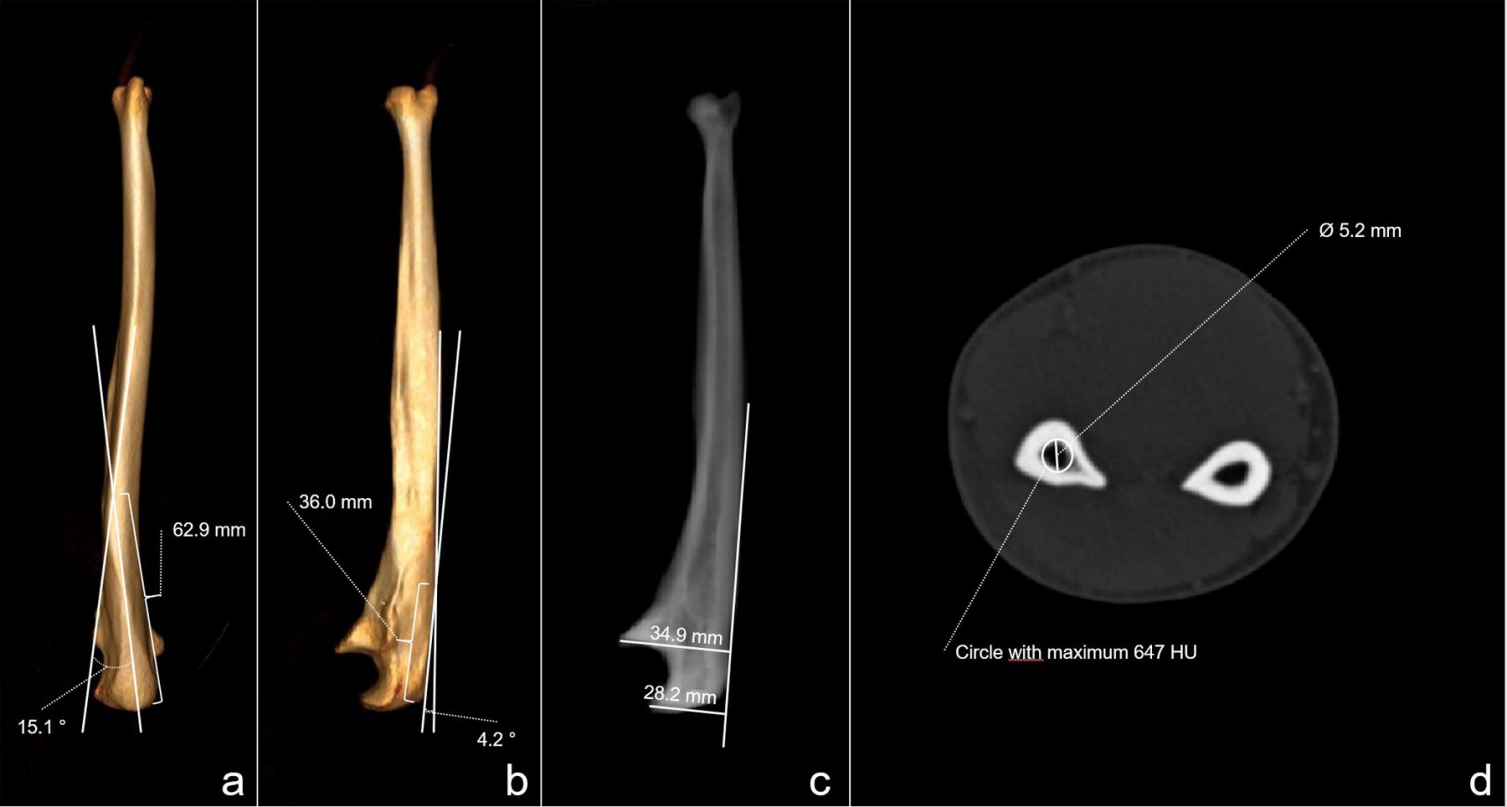
Morphometric measurements. a: Measurements of the proximal ulna varus angulation and apex of varus angulation; b: Proximal ulna dorsal angulation (PUDA) and apex of PUDA; c: Coronoid and olecranon heights; d: Diameter of the medullary canal in a 2d-slice with a maximum Hounsfield unit of 647.

All measurements were done with Sectra Workstation IDS 7 (Sectra Medical, Linköping, Sweden). The proximal ulna dorsal angulation (PUDA) and varus angulations were measured in a 3-dimensional reformation of the ulna in strict lateral and dorsal views. The varus angulation was measured as the angle between the axis of the proximal ulna and the axis of the ulna shaft (Fig 1A). The dorsal angulation was measured as the angle between the tangents of the proximal ulna and the ulna shaft (Fig 1B). The coronoid and olecranon heights were measured after a multiplanar reformation in a 2-dimensional image in strict lateral view of the ulna bone perpendicular to the posterior surface of the ulna. The last measurements were done from the posterior cortex to the tip of the process (Fig 1C). The location of the dorsal and varus angulation was defined in the 3-dimensional image as the point of intersection of tangents to the proximal ulna and ulna shaft like described for cadaveric ulnae (Fig 1A and 1B) [6,15]. The length of the ulna was measured from the olecranon tip to the distal surface of the ulna without measuring the ulnar styloid process in a 3-dimensional image of the ulna bone.

The medullary canal was visualized in 2-dimensional axial slices after exact multiplanar reformations along the ulnar axis. The multiplanar reformations were done perpendicular to the axis of the ulna shaft. A threshold of 700 Hounsfield units was defined for cortical bone according to the available literature [22,23]. The maximal diameter of the medullary canal was measured every 10mm in axial slices of the ulna starting 50mm from the olecranon tip until the distal surface of the ulna (Fig 1D). With the ROI tool (region of interest) the greatest possible circle was placed into the medullary canal in the axial slice without exceeding the circle into bone with a density over 700 Hounsfield units. The first 10% of the patient cohort were measured by two authors. No significant differences were found between both researchers. The following measurements were done only by one researcher. On a random basis values were double-checked by both authors. Again, no relevant differences were found.

### Statistical analysis

For all statistical calculations, R version 3.3.0 (R Foundation for Statistical Computing, Vienna, Austria) was used and all diagrams were created using R or the ggplot2-package (H. Wickham et al., Springer, New York) within R. Univariate means, standard deviations and quantiles were calculated to assess the central tendency and spread of all measurements. Estimated Normal Distribution density functions were added to histograms for easier visual assessment of the empirical distribution. Linear regression lines with 95% confidence intervals were added to scatterplots for the visual assessment of linear relationships. Bivariate relationships were assessed by calculating Pearson’s and Spearman’s Rank Correlation Coefficient or by fitting simple linear regression models for pairs of variables. For correlation coefficients, a value ≥ 0.7 was considered a strong correlation; ≥ 0.3 was considered a moderate correlation; ≥ 0.1 a weak correlation; and < 0.1 no correlation [24]. For exploratory hypothesis tests based on linear regression models, p-values < 0.05 were considered as significant.

### Ethics statement

The analysed data was fully anonymized before data assessment. No informed consent was needed for the execution of this study. The analysed data in this study is part of the doctoral thesis of A. Jaehnig.

## Results

### Proximal ulna

**Fig 2:**
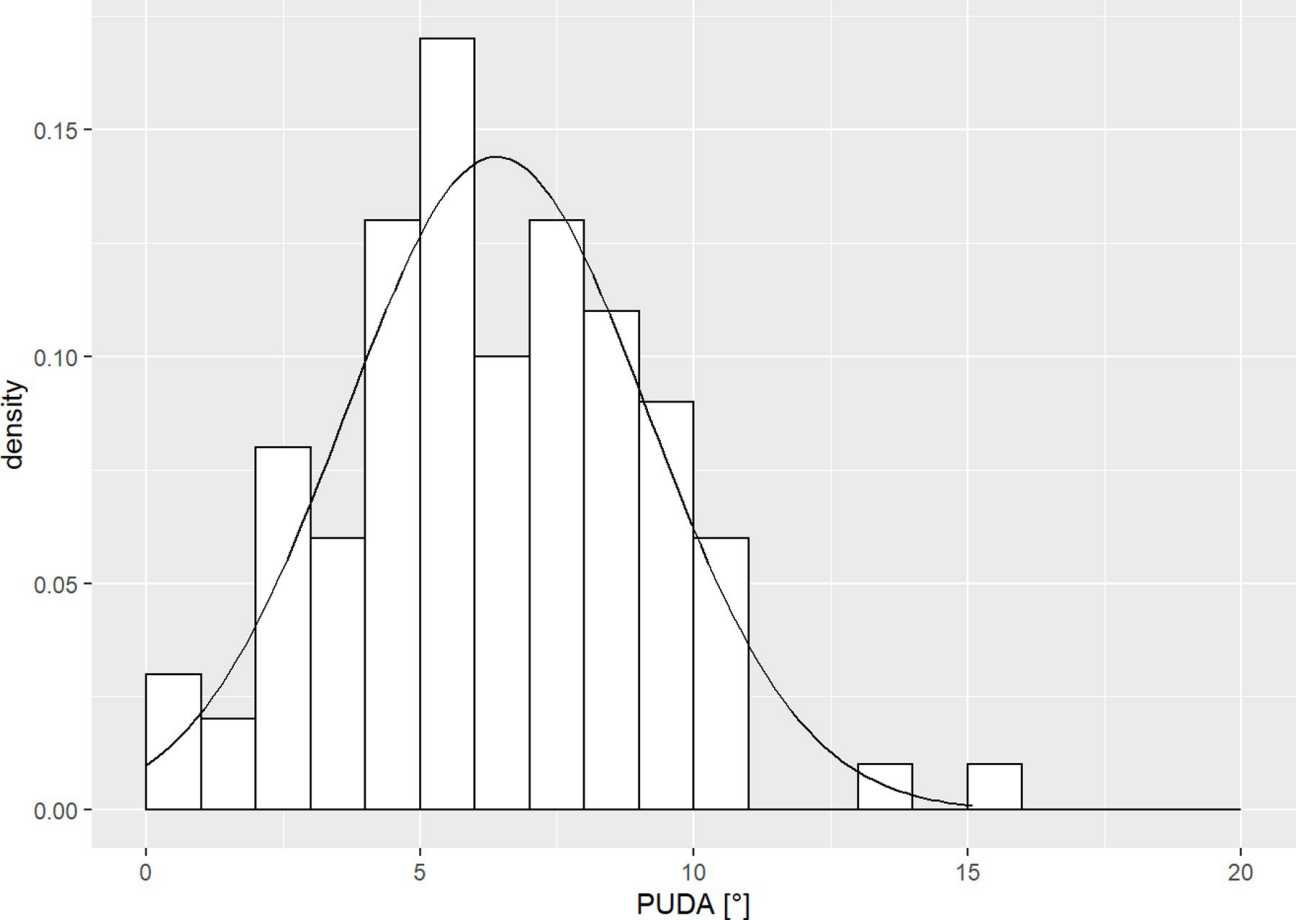
PUDA histogram. Histogram of the proximal ulna dorsal angulation (PUDA) of our patient cohort.

**Fig 3:**
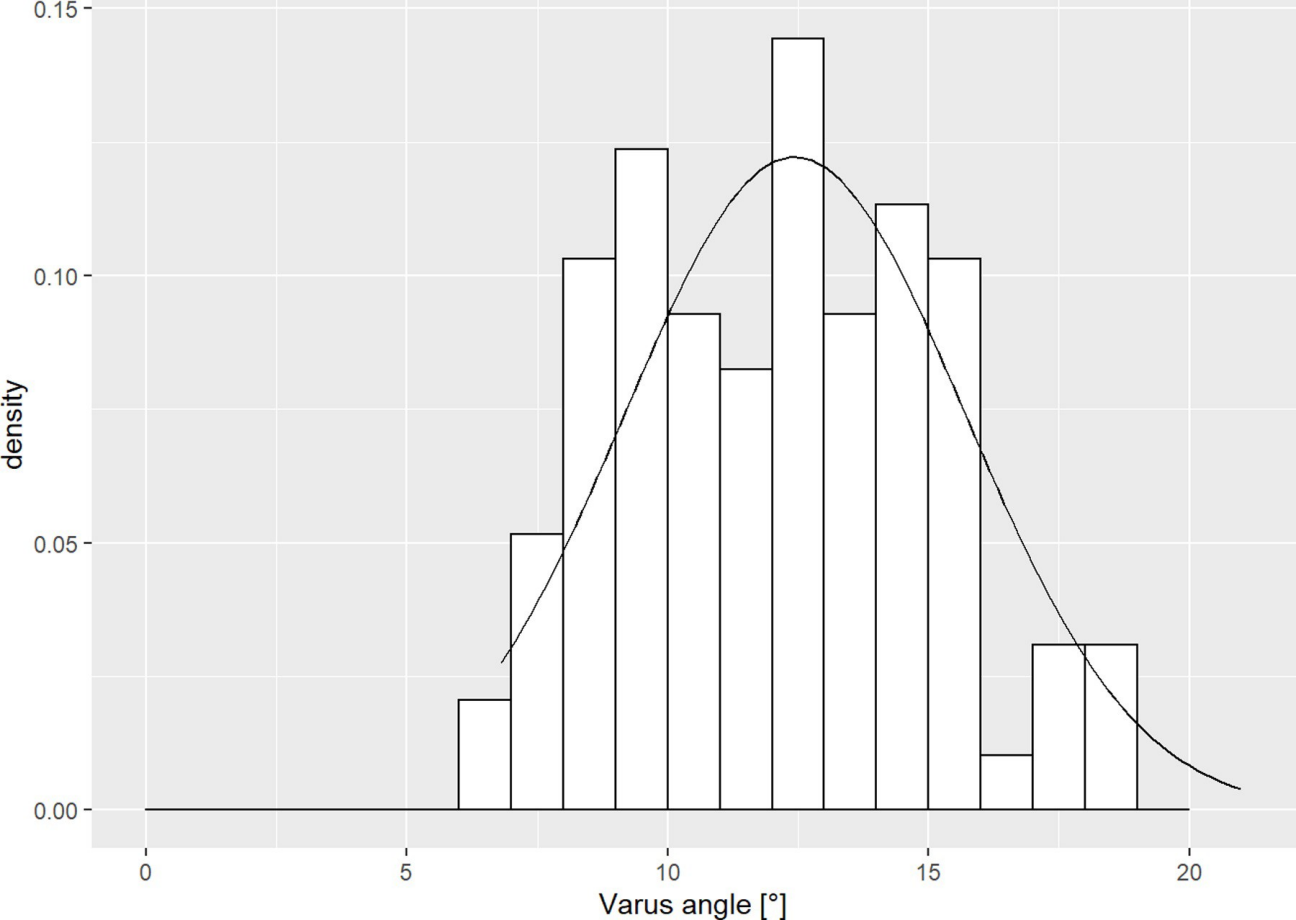
Varus angulation histogram. Histogram of the proximal ulna varus angulation of our patient cohort.

The average proximal ulna dorsal angulation (PUDA) was 6.4° (SD 2.8°), the apex located at 58.9 mm (SD 20.2 mm) from the olecranon tip (Fig 2). The proximal ulna varus angulation was 12.4° (SD 3.3°), the apex at 47.8 mm (SD 10.3 mm) from the olecranon tip (Fig 3). The mean coronoid height was 36.1 mm (SD 3.9 mm), while the mean olecranon height amounts 23.6 mm (SD 3.2 mm).

### Ulna diaphysis

**Fig 4:**
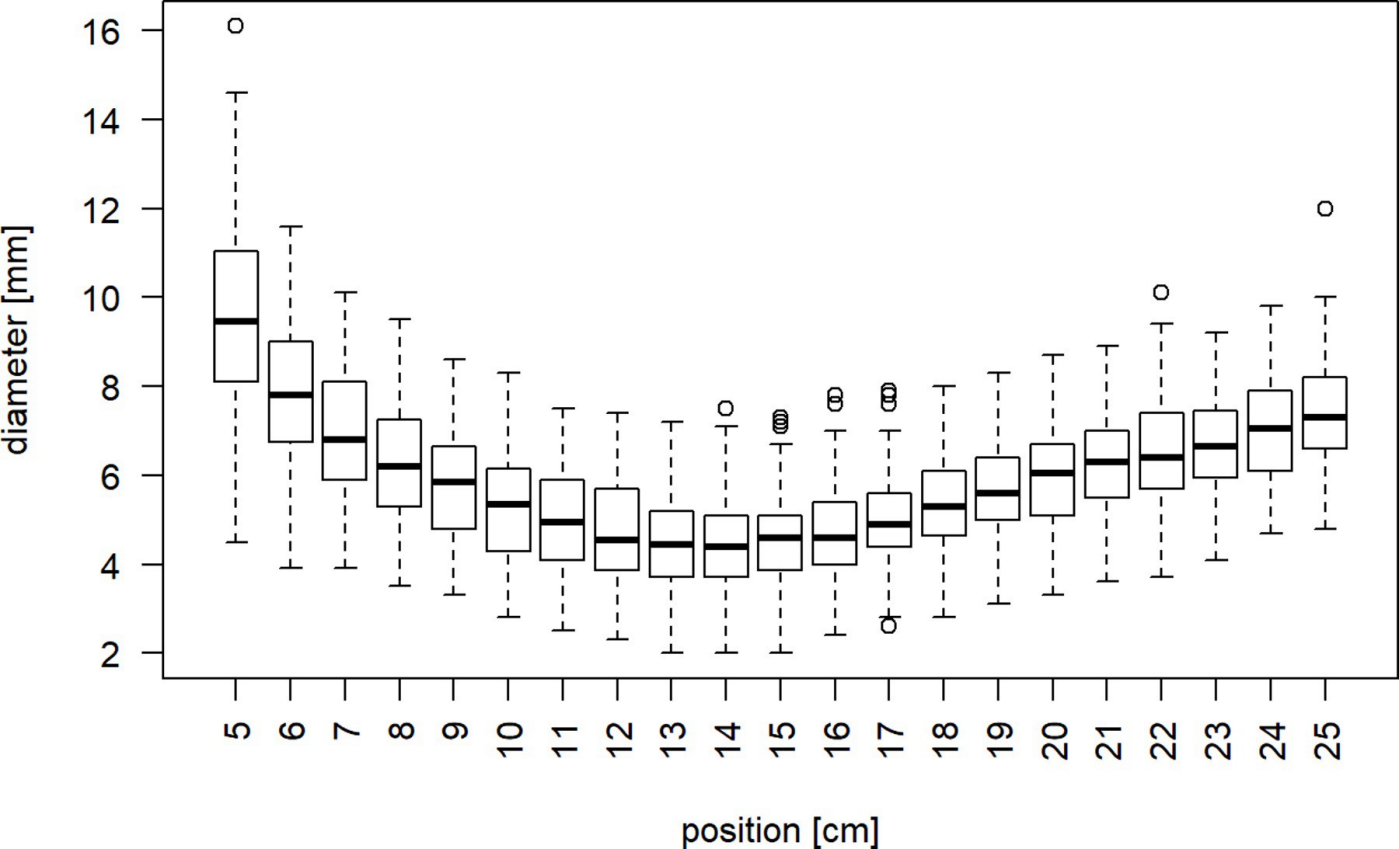
Diameter of medullary canal. Distribution of the maximum diameter of the medullary canal depending on the distance from the olecranon tip in boxplots.

**Fig 5:**
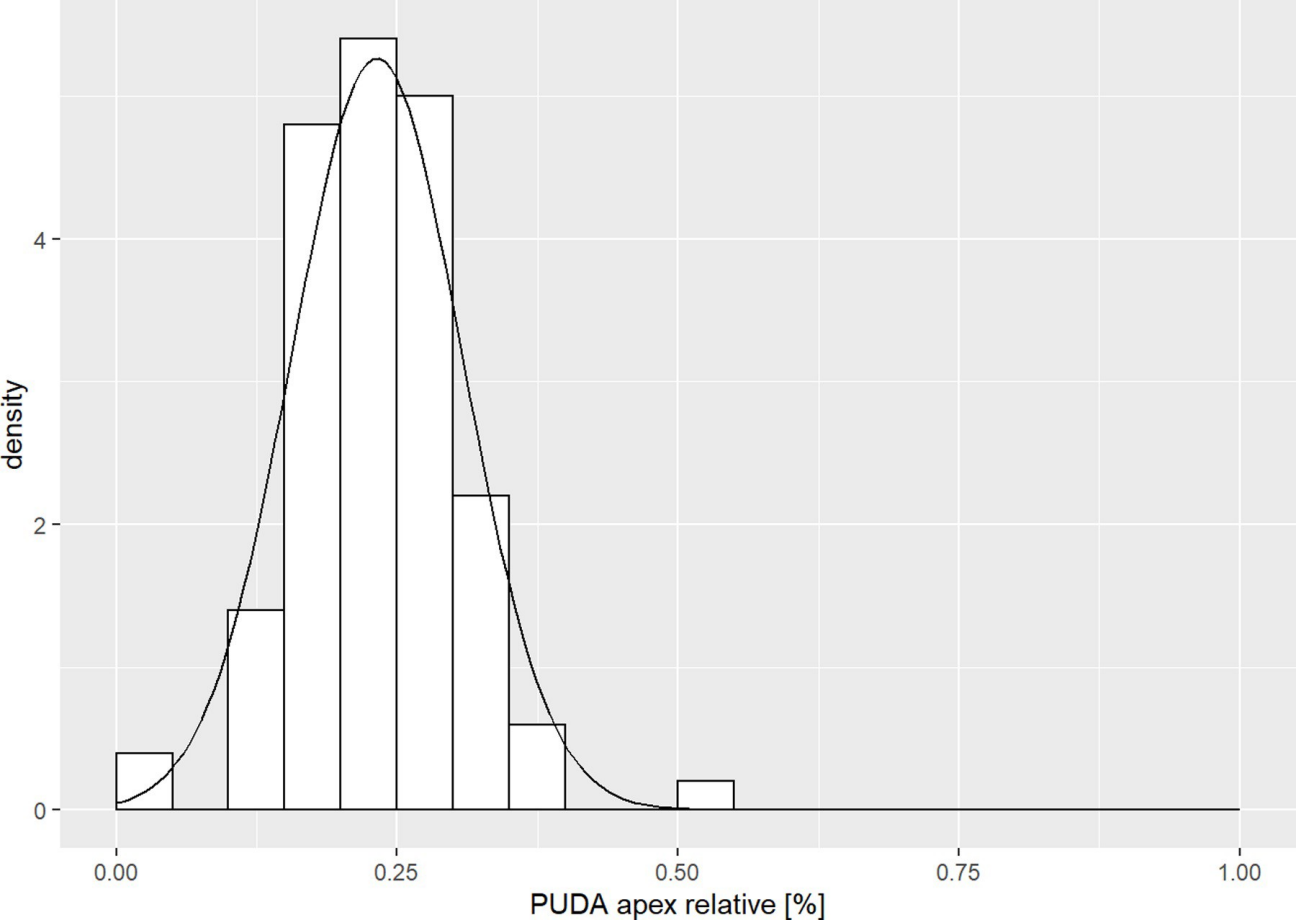
PUDA in relation to ulnar length. Histogram of the PUDA apex in relation to the total length of the ulna.

The smallest diameter of the medullary canal was 4.2 mm (SD 1.1 mm) located at 141.3 mm (SD 19.7 mm) from the olecranon tip (Fig 4). 56% of the patient cohort had a minimum diameter larger than 40mm, 21% of the patients larger than 50mm. The mean length of the ulna was 253.6 mm (SD 19.9 mm). Our analyses showed a weak correlation (Pearson r = 0.26) of the apex of the PUDA and the length of the ulna. The apex was approximately normally distributed with the median at 23.2% (SD 0.1) of the total length of the ulna (Fig 5). The mean relative position of the apex of the varus angulation was at 18.9% (SD 0.1) of the length of the ulna without a correlation of both parameters (Pearson r = 0.1).

**Fig 6:**
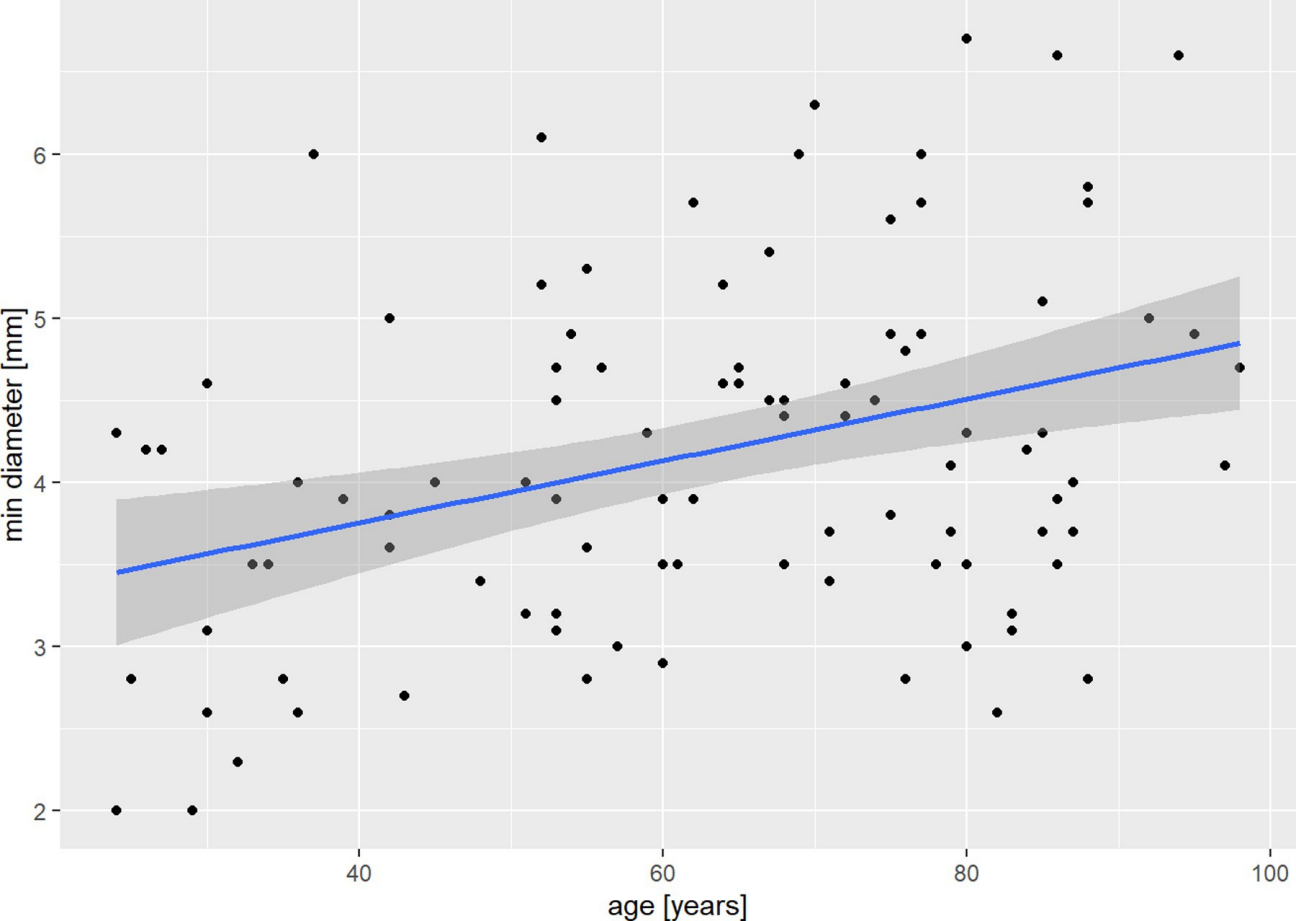
Diameter of medullary canal and age. Scatterplot of the correlation of minimum diameter of the medullary canal and age in our patient cohort with regression line and estimated confidence interval.

**Fig 7:**
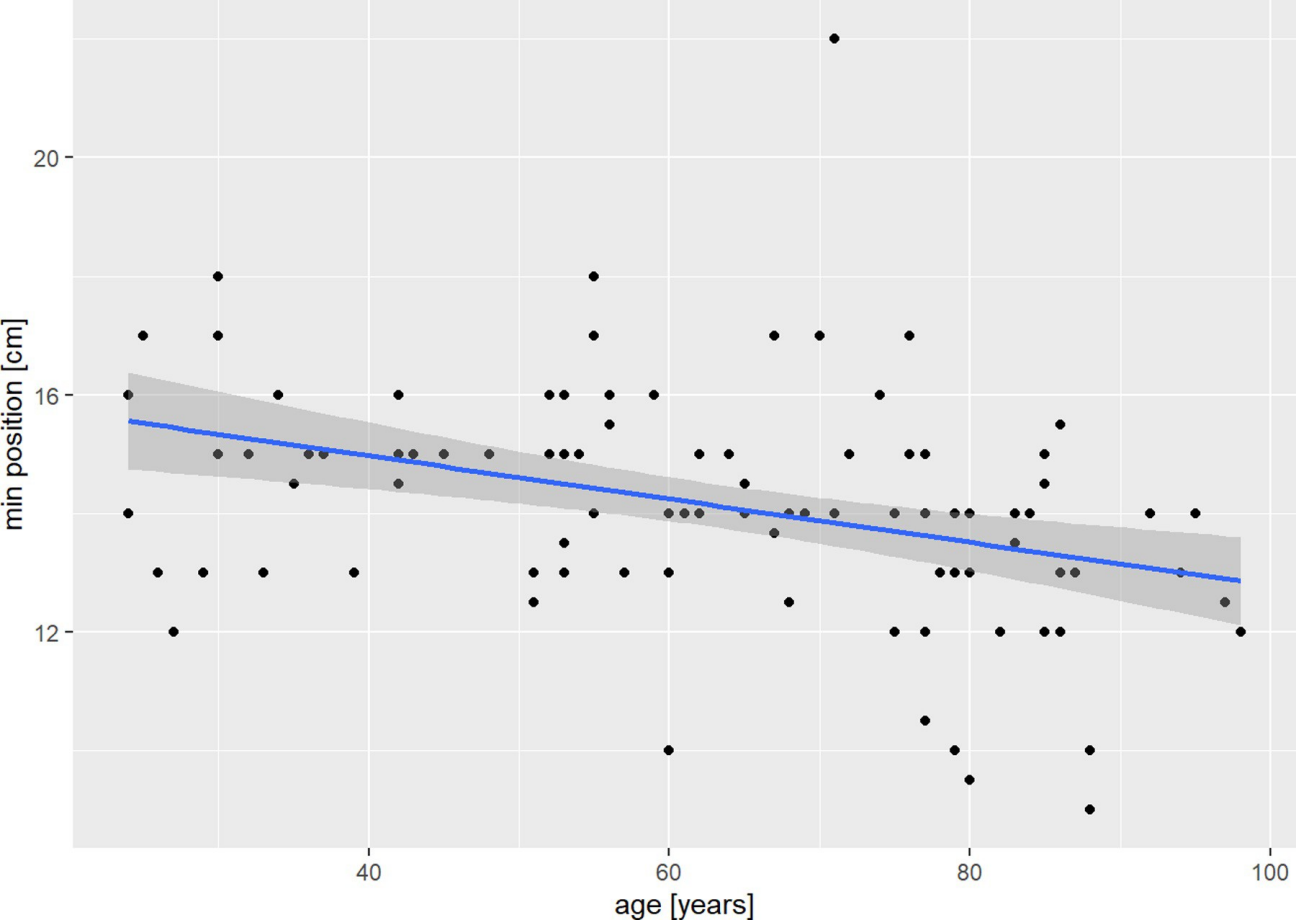
Position of minimum diameter and age. Scatterplot of the correlation of the position of the minimum diameter of the medullary canal and age in our patient cohort with regression line and estimated confidence interval.

The minimal diameter of the medullary canal correlated weakly with increasing age (Spearman r = 0.3; Fig 6). The position of the minimum diameter of the medullary canal showed a moderate negative correlation with increasing age (Spearman r = -0.4; Fig 7). Also, the length of the ulna showed a moderate negative correlation with increasing age (Spearman r = 0.41). PUDA and apex of PUDA, varus angulation and apex of varus angulation, the coronoid and olecranon height showed no correlation with age.

In our subgroup analysis PUDA and varus angulation were independent of sex. Other measurements like ulnar showed a significant difference in both sexes (Table 2).

**Table 2:** Subgroup analysis.

Parameters	Gender	Mean	SD	Min	Max	p-value (t-test)
PUDA [°]	Male	6.4	2.7	0	10.9	0.89
	Female	6.4	3.0	0	15.1	
	Total	6.4	2.8	0	15.1	
Apex of PUDA [mm]	Male	63	21.9	0	140.1	0.01*
	Female	52.6	15.5	0	78.6	
	Total	58.9	20.2	0	140.1	
Varus angulation [°]	Male	12.5	3.3	7	21	0.76
	Female	12.3	3.2	6.8	21	
	Total	12.4	3.3	6.8	21	
Apex of varus angulation [mm]	Male	47.8	10.2	23.7	71	0.99
	Female	47.9	10.6	29.3	70	
	Total	47.8	10.3	23.7	71	
Minimal diameter of medullary canal [mm]	Male	4.2	1	2	6.3	0.66
	Female	4.1	1.2	2	6.7	
	Total	4.2	1.1	2	6.7	
Position of minimal diameter [mm]	Male	146.8	20	95	220	< 0.01*
	Female	133.2	16.3	90	160	
	Total	141.3	19.7	90	220	
Coronoid height [mm]	Male	37.7	3.2	28.3	44	< 0.01*
	Female	33.6	3.5	24.9	44	
	Total	36.1	3.9	24.9	44	
Olecranon height [mm]	Male	24.8	2.8	17.2	30.6	< 0.01*
	Female	21.9	3.0	17.1	33	
	Total	23.6	3.2	17.1	33	
Ulnar length [mm]	Male	264.9	14.3	217.8	295	< 0.01*
	Female	236.7	14.2	202.4	257.2	
	Total	253.6	19.9	202.4	295	

Subgroup analysis of the CT-based measurements of the ulna

* p<0.05 was considered as statistically significant

## Discussion

We described the anatomy of the ulna with special attention to the configuration of the proximal ulna using a large cohort of 100 individual bones based on CT scans. Information about the relevant angles and proportions of the proximal ulna as well as the diameter of the medullary canal of the ulna shaft were provided and statistically analysed.

The intention of this study was to generate an anatomical base for the development of a new ulna nail for fixation of shaft fractures. Especially in comminuted fractures or open fractures of the ulna, a load-bearing intramedullary implant may be advantageous [18,19]. Intramedullary implants have to adapt to the complex anatomy of the proximal ulna with a varus configuration and a dorsal angulation. Even anatomically preshaped plates have to be bent often intraoperatively to match the anatomy of the proximal ulna [14]. Morphological properties of the proximal ulna and the entry point chosen at the olecranon have a high impact on the fit of the nail in the ulna bone, especially for non-anatomic shaped nails [25]. Geometrical characteristics of a newly designed short ulna nail for the treatment of proximal ulna fractures were described in detail in a recent biomechanical study [20].

Our measurements of the ulnar length and the angulations of the proximal ulna are consistent to existing cadaveric and computed tomography studies of the ulna [5,8,13–15]. The mean PUDA is more consistent (4.3° - 8.5°) than the proximal varus angulation (8.5° - 17.5°), which shows a larger range in the literature (Table 3). Also the results of the coronoid height and olecranon height are similar to existing studies, which used the same measurement method [6] (Table 3). The apex of the varus angulation in our study is more proximal compared to the existing literature (Table 3). This could be due to a lack of standardisation of the measurement methods. We found an average ratio of the PUDA apex to the total ulnar length of 23.2% which is comparable to the CT-analysis from Yong et al (26.4%) [5]. Two recent studies investigated the anterior-posterior and lateral bow of the ulna bone and published slightly different results compared to the existing literature [26,27]. Especially the location of the ulna bows were described to be more distally than presumed previously [26,27]. It must be noted, that the comparison between different studies are impeded because of the different measurement methods.

**Table 3:** Comparison of ulna measurements.

Study	n	PUDA [°]	Varus angulation [°]	Apex of varus angulation [mm]	Ulnar length [mm]	Coronoid height [mm]	Olecranon height [mm]
Own study	100	6.4 ± 2.8	12.4 ± 3.3	47,8 ± 10,3	253.6 ± 19.9	36.1 ± 3.9	23.6 ± 3.2
Beşer et al. [6]	50	8 ± 2.3	9.3 ± 2.2		250.5 ± 14.9	33.1 ± 2.4	24.6 ± 2.6
Grechenig et al. [13]	54		17.5	73.2	262		
Puchwein et al. [14]	40	6.2 ± 2.7	14.3 ± 3.6	75 ± 7.9			24.7 ± 2.7
Totlis et al. [15]	100 pairs	8.5 ± 2.7	8.5 ± 2.6	81.9 ± 12.6			
Wang et al. [8]	20 pairs			76	260		
Yong et al. [5]	20	4.3 ± 0.9	12.1 ± 2.6			35.1 ± 0.9 (SE)	

Mean value and standard deviation of proximal ulna dorsal angulation (PUDA), varus angulation, ulna length, coronoid and olecranon height found in our study, compared to findings in the literature; SE: standard error

The diameter of the medullary canal is difficult to compare with existing studies due to the different measurement methods [3,28]. We demonstrated the narrowest medullary canal being at 14.1 cm with enlarging thereafter again. The increasing minimal diameter with age may be explained by increasing inner cortical porosity with decreasing cortical thickness due to osteoporosis [29]. In patients with rheumatoid arthritis, the canal was non-significantly smaller in this area compared to cadaveric bone [28].

The dimensions of the ulna were smaller in females than in males. However, as the body height was not available, we cannot conclude if they depend on gender or on body dimensions.

The methods we used with manual measurement of 2- and 3-dimensional reformations of forearm CT scans may be vulnerable to examiner-related variances. Other morphometric analyses of ulna measurements were done automatically using a computer-aided-design software, aiming to a higher reproducibility [3,5]. However, yet published studies based on CT data of cadaveric bones used considerably smaller sample sizes [3,5,7]. As we aimed to describe anatomic variables to develop an implant for fracture fixation, our collective represented the gender and age distribution of the fracture cohort.

To reduce examiner-related differences, the research method was defined precisely and measurements performed by only one researcher. Still, the manual data acquisition and the assessment by one observer only can be considered as a limitation of our study. The use of computed tomography for image acquisition may lead to discrepancies compared to cadaveric bone morphometrics due to image processing errors. The threshold of 700 Hounsfield units for the corticocancellous interface is an empirical chosen value, which may not exactly correlate with the density of cortical bone in living humans [22].

An anatomic analysis of the same patient collective with statistical shape modeling as described for other bones is planned to validate our manually acquired results [30–32]. Based on the acquired data, a novel intramedullary anatomically preshaped implant could be developed and successfully tested in a biomechanical study and on cadaveric bones. When designing an implant for the ulna shaft, especially the higher varus angulation has to be considered. As the inner cortical diameter can be as small as 2mm, reaming would be necessary prior to nail insertion. Biomechanically, the developed nail can be regarded as an alternative implant to plate fixation especially for the stabilisation of comminuted fractures [20].

We conclude, that knowledge of the relevant anatomic parameters of the ulna is important for exact restoration of the anatomy and fracture fixation. Our study provides a novel description of the complex anatomy of the ulna bone in a representative patient cohort. Also, correlation between the measurements and age and sex could be evaluated. The knowledge of these anatomic parameters can lead to an improvement of implant design for the fixation of proximal and diaphyseal ulna fractures.

## Supporting information

S1 Raw data (XLSX)
